# Treatment of Burns

**Published:** 1848-01

**Authors:** 


*Treatment of Burns.*—M. Jobert, of the Hôpital St. Louis, has tried the prolonged application of ice to severe burns, and this with the most happy results. Two cases occurring recently in his practice are briefly recorded in *L'Union Médicale.* A man was brought into the hospital with a deep and extensive burn of the right hand—a burn so bad, that the two last phalanges of the index and middle fingers sloughed and required amputation. The other fingers were much less deeply injured, but yet very severely; the calf also presented a large scar. M. Jobert applied lint spread with cerate, immediately to the wounds, and over this placed some bladders filled with ice, which were kept constantly applied, the ice being frequently renewed. No febrile reaction took place; the sloughs separated, and cicatrization went on rapidly, without the formation of bands to contract. The other case was still more severe, and occurred in a young man employed in a match-factory, who was burned by the deflagration of a mass of phosphorus which he was preparing. The entire face was burned; the globe of the right eye, including the cornea itself, was covered by a burnt scab, which, on separating, has left a large ulcerating surface, suppurating copiously; the forehead, nose, and cheeks, were also implicated in the burn. The use of the ice, in the preceding manner, has prevented the development of any of those inflammatory accidents which are so common in burns so extensive; febrile reaction has been scarcely sensible; the pulse hardly increased in frequency; and the pain very moderate. There has been, however, a little delirium, but connected, according to M. Jobert, not with the general state of the system, but with the local inflammatory action going on in the eye. Cicatrization has proceeded quickly, and no cellulo-cutaneous bands have formed; the new skin formed is smooth and even, and the production of new tissues has taken place with perfect regularity.—*Ib.*

